# The effect of freeze-dried *Carica papaya* leaf juice treatment on NS1 and viremia levels in dengue fever mice model

**DOI:** 10.1186/s12906-018-2390-7

**Published:** 2018-12-05

**Authors:** Mohd Ridzuan Mohd Abd Razak, Norazlan Mohmad Misnan, Nur Hana Md Jelas, Nor Azrina Norahmad, Amirrudin Muhammad, Tiffiny Chau Dee Ho, Bazilah Jusoh, Umi Rubiah Sastu, Murizal Zainol, Mohd Isa Wasiman, Hussin Muhammad, Ravindran Thayan, Ami Fazlin Syed Mohamed

**Affiliations:** 1 Herbal Medicine Research Center, Institute for Medical Research, Kuala Lumpur, Malaysia; 2 Infectious Disease Research Center, Institute for Medical Research, Kuala Lumpur, Malaysia

**Keywords:** Dengue, AG129 mice, *Carica papaya* leaf, nonstructural protein 1, viremia

## Abstract

**Background:**

*Carica papaya* leaf juice (CPLJ) was well known for its thrombocytosis activity in rodents and dengue patients. However, the effect of CPLJ treatment on other parameters that could contribute to dengue pathogenesis such as nonstructural protein 1 (NS1) production and viremia level have never been highlighted in any clinical and in vivo studies. The aim of this study is to investigate the effect of freeze-dried CPLJ treatment on NS1 and viremia levels of dengue fever mouse model.

**Methods:**

The dengue infection in mouse model was established by inoculation of non-mouse adapted New Guinea C strain dengue virus (DEN-2) in AG129 mice. The freeze-dried CPLJ compounds were identified by Ultra-High Performance Liquid Chromatography High Resolution Accurate Mass Spectrometry analysis. The infected AG129 mice were orally treated with 500 mg/kg/day and 1000 mg/kg/day of freeze-dried CPLJ, starting on day 1 post infection for 3 consecutive days. The blood samples were collected from submandibular vein for plasma NS1 assay and quantitation of viral RNA level by quantitative reverse transcription PCR.

**Results:**

The AG129 mice infected with dengue virus showed marked increase in the production of plasma NS1, which was detectable on day 1 post infection, peaked on day 3 post-infection and started to decline from day 5 post infection. The infection also caused splenomegaly. Twenty-four compounds were identified in the freeze-dried CPLJ. Oral treatment with 500 mg/kg/day and 1000 mg/kg/day of freeze-dried CPLJ did not affect the plasma NS1 and dengue viral RNA levels. However, the morbidity level of infected AG129 mice were slightly decreased when treated with freeze-dried CPLJ.

**Conclusion:**

Oral treatment of 500 mg/kg/day and 1000 mg/kg/day of freeze-dried CPLJ at the onset of viremia did not affect the plasma NS1 and viral RNA levels in AG129 mice infected with non-mouse adapted New Guinea C strain dengue virus.

**Electronic supplementary material:**

The online version of this article (10.1186/s12906-018-2390-7) contains supplementary material, which is available to authorized users.

## Background

Globally, up to 390 million dengue infections are estimated to occur yearly and 3.9 billion people are at risk of infection with dengue virus in over 128 endemic countries [1]. The person infected with dengue virus may develop a flu-like illness called febrile dengue and may progress into a lethal complication called severe hemorrhagic dengue [1]. The dengue virus is transmitted by female *Aedes* mosquito from infected human to other healthy human [1]. The virus comprises 4 serotypes, DEN-1, DEN-2, DEN-3 and DEN-4 which belong to the genus *Flavivirus*, family Flaviviridae [1]. To date, there is no specific anti-viral drugs for dengue treatment as most of the potential candidates failed to reduce viremia and antigenemia levels in clinical studies [2]. Although the dengue vaccine such as Dengvaxia is available, the efficacy of this vaccine is limited to seropositive individuals or individuals who have been infected with dengue [3].

In a descriptive cross-sectional study conducted in the hospital setting in Malaysia, CPLJ was among the popular complementary alternative medicine used by the patients with dengue fever after isotonic drinks and crab soup [4]. Indeed, the thrombocytosis effect of CPLJ have been previously documented in several clinical trials and case studies conducted on dengue patients [5–12]. The thrombocytosis activity of the *Carica papaya* leaf juice (CPLJ) and extracts have been scientifically proven *in vivo* either in healthy rodents or thrombocytopenic induced rats [13–19]. In addition, the treatment of *C. papaya* leaf aqueous extract has been shown to decrease the bleeding and clotting time in thrombocytopenic induced rats [13]. The actual mechanism of thrombocytosis activity induced by CPLJ is still unknown. The potential compounds that exhibited the thrombocytosis activity could be derived from the alkaloids [19], phenolics and flavonoids which were majorly found in CPLJ [13, 16].

Besides the thrombocytosis effect, other factors related to dengue severity or pathogenesis such as viremia and NS1 antigenemia developments have not been addressed in any *in vivo* and clinical studies involving CPLJ. Dengue virus NS1 contributed to dengue pathogenesis by inducing plasma leakage [20] through the activation of apoptosis [21], Toll-like receptor 4 [22] and expression of sialidases and heparanase from the endothelial cells, which could disrupt the endothelial glycocalyx layer [23]. It was also reported to be involved in thrombocytopenia by exhibiting autoantibody production, which caused the platelet phagocytosis by the macrophages [24] during dengue virus infection. Therefore, the NS1 level could potentially be used as a marker for dengue severity and any efforts in perturbing the NS1 production either by affecting the virus replication or host mediated NS1 secretion pathways may potentially reduce the burden of dengue patients. In addition, the potential of quercetin, a compound presence in CPLJ, to inhibit the dengue viral replication has been demonstrated by in silico [25] and in vitro [26] studies.

The AG129 mouse, lacking the interferon α/β and γ receptors, was first reported as a reliable dengue mouse model by Johnson and Roehrig in 1999 [27] after establishing the infection with a mouse-adapted DEN-2 virus strain, New Guinea C (NGC) [27]. In human infection, dengue virus replicates successfully by inhibiting interferon signaling. However, the inhibition of interferon signaling by dengue virus was not successful in mouse [28]. So, with the absence of interferon α/β and γ receptors, the immune response related to interferon signaling was impaired. Hence, the AG129 mouse become susceptible to viral infection such as dengue, vaccinia, lymphocytic choriomeningitis [29] and Zika viruses [30]. The AG129 mouse was commonly used as a dengue fever and dengue hemorrhagic in vivo models in many preclinical drug discovery studies [31–34]. Depending on dengue virus strain, the infected AG129 mice could mimic several major pathologies of human dengue infection such as high level of viremia, overproduction of proinflammatory cytokines, vascular leakage and thrombocytopenia [35–38]. Thereby, this study has established the dengue fever mouse model by inoculation of non-mouse adapted DEN-2 dengue virus strain, NGC, which was used as a test system to study the effect of freeze-dried CPLJ treatment on NS1 antigenemia and viremia developments.

## Methods

### Animal ethics statement and husbandry

All animal experiment procedures were carried out under the guidelines and upon approval of the Animal Care and Use Committee, Ministry of Health Malaysia (ACUC-MOH), (ACUC/KKM/02(9/2016). The AG129 mice (129/Sv mice deficient in both alpha/beta and gamma interferon receptors) (5-6 weeks old) were obtained from Marshall BioResources, United Kingdom. All experiments were conducted in the GLP-certified Non-Clinical Research Facility, Laboratory Animal Research Unit, Medical Research Resource Center, Institute for Medical Research, Kuala Lumpur, Malaysia. The animals were received artificial light, 12 hours light and 12 hours dark. The experimental room’s temperature was maintained within the range 19°C to 26°C. All experiment procedures on mice were conducted by trained personnel and assisted by the veterinarian. The mice were housed in individual ventilated cages supplied with reverse osmosis drinking water and mouse pellet *ad libitum*. Prior to the experiment, the mice were quarantined for 2 weeks and acclimatized for 1 week.

### Experimental design

The experiments were carried out independently in two phases, the establishment of dengue fever mouse model and the efficacy evaluation of freeze-dried CPLJ. For the establishment of dengue fever model in AG129 mice, 7 to 8 weeks old male mice were randomly assigned into four groups comprised of one mock infected group (inoculated with plain media) (*n*=5) and three infected groups. The mice in each of infected group were inoculated with 2X10^3^ PFU (*n*=5), 2X10^4^ PFU (*n*=5) and 2X10^5^ PFU (*n*=5) of dengue virus. The whole bloods (~250 μl) were collected through submandibular vein on day 1, 3, 5, 7 and 9 post infections for NS1 assay and viral RNA quantitation as mentioned in the following sections. All mice were euthanized on day 11 post infection. For this phase of experiment, the higher inoculation titer (2X10^6^ PFU) was not tested due to the limited titer of virus stock. There are several factors or conditions that influenced the production of virus stock with higher titer such as culture conditions, timing of virus harvesting, choice of host cell types and virus strains [39–41]. The virus harvesting in this study was based on the observable cytopathic effect (CPE) of infected host cells, which seem to result in limited titer of virus stock. However, virus harvesting at the exponential phase of viral production, after kinetic evaluation, has led this study to obtain a higher virus titer for the second phase of the experiment.

For the efficacy evaluation of freeze-dried CPLJ, the mice were inoculated with 2X10^5^ PFU intraperitoneally. In addition, an independent experiment was done on mice infected with higher inoculation titer, 2X10^6^ PFU, which produced higher lethality rate than the mice infected with 2X10^5^ PFU dengue virus (Additional file 1). The AG129 mice were randomly assigned into 4 groups comprised of mock infected group (mock infected + distilled water) (*n*=5), treatment control group (mock infected + 1000 mg/kg BW of CPLJ) (*n*=5), infected group (infected + distilled water) (*n*=5) and treatment group (infected + 500 mg/kg BW of CPLJ) (*n*=5). The morbidity level of each mice was observed and monitored as mentioned in previous section for 30 days post infection. In addition, another independent experiment was done to evaluate higher treatment dose of freeze-dried CPLJ, 1000 mg/kg BW. For this experiment, three groups of mice; mock infected group (*n*=5), infected group (*n*=5) and treatment group (*n*=5) were used. The preparation of freeze-dried CPLJ for dosing is as mentioned in the following section.

### Cell and dengue virus propagation

The mosquito C6/36 and monkey kidney Vero cells were purchased from American Type Culture Collection (ATCC, USA). C6/36 cells were grown at 28°C in Leibovitz’s L-15 medium supplemented with 2% FBS, 10% tryptose phosphate broth, 100 U/ml penicillin G and 100 μg/ml streptomycin. Vero cells were grown in DMEM medium supplemented with 10% fetal bovine serum, 35 mM sodium bicarbonate, 100 U/ml penicillin G and 100 μg/ml streptomycin at 37°C in 5% CO_2_ Galaxy 170 S incubator (New Brunswick, Germany).

The DEN-2 dengue virus stock, New Guinea C strain (NGC) (ATCC VR-1584) was grown in C6/36 cells at 28°C in Leibovitz’s L-15 medium. The titer of dengue virus was quantified by plaque assay. Briefly, 4.5 X 10^5^ of Vero cells were grown in a 6-well plate containing 1.5 ml DMEM medium for 24 hours until confluent. Then, the media was discarded and replaced with 100 μl of C6/36 cell supernatant containing virus suspension. The viruses were allowed to infect the Vero cells for one hour. One ml of an overlay medium containing 1% agarose in 1X DMEM medium was added to Vero cell monolayers and incubated for 7 days at 37°C in 5% CO_2_ incubator. The Vero cell monolayers were stained with 1% crystal violet in 20% ethanol leaving clear unstained plaques resulted from virus infection. The infectivity titre was expressed as the number of plaque forming units per ml (PFU/ml).

### Dengue virus inoculation and morbidity monitoring

The AG129 mice were inoculated with DEN-2 dengue virus strain, NGC, via intraperitoneal route according to the titers mentioned in previous section. At 24 hours post infection, the body weight of the mice was recorded daily. The sign of illness was monitored twice a day and scored based on 1 to 5 scale: 1-healthy; 2-mild sign of lethargy and ruffled fur; 3-intermediate level of lethargy, ruffled fur and hunched posture; 4-very lethargy, ruffled fur, hunched posture and limited mobility; 5-moribund with limited to no mobility and inability to reach food or water [42]. Mice exhibiting weight loss more than 20% of initial body weight or moribund or paralyzed during the study were euthanized immediately by open-drop exposure to isoflurane. Euthanized mice during the study were counted as being dead on the following day for analysis [35]. At the end of the study, the survived mice were euthanized by open-drop exposure to isoflurane by the veterinarian in the fume hood. None of the mice died before meeting criteria for euthanasia.

### Freeze-dried *C. papaya* leaf juice preparation

Fifty grams of *C. papaya* fresh green leaves from ‘Sekaki’ cultivar, were collected from the herbal garden of the Institute for Medical Research, Malaysia. The *C. papaya* was identified by Ms. Tan Ai Lee, a botanist from the Forest Research Institute of Malaysia (FRIM). The voucher specimen was deposited in the FRIM, Kepong, Malaysia (Voucher No: 007/10). The *C. papaya* trees were planted organically without the use of pesticides and herbicides. Only healthy leaves (without ring spot) were selected for the juice preparation. The leaves were cleaned thoroughly with veggie wash (to remove wax and soil) followed by washing with reverse osmosis water and extracted using Panasonic juicer Mj68 (Shah Alam, Malaysia) without any addition of water in a biosafety cabinet class II. The remaining husk was pressed using a clean laboratory cloth. The juice was then placed in a sterile container and frozen at -50°C before subjected to freeze drying process in a freeze-dryer Alpha 1-2 LD Plus (Martin Christ, Germany) to obtain the powdered form of the juice, freeze-dried CPLJ.

### Ultra-High Performance Liquid Chromatography-High Resolution Accurate Mass Spectrometry (UHPLC-HRAMS) analysis

The following reference standards were purchased from Extrasynthese (Genay, France): quercetin-3-O-rutinoside (rutin), kaempferol-3-O-rutinoside (nicotiflorin), 1,2-dihydroxybenzene (catechol), ferulic acid, morin and isorhamnetin. Kaempferol, 5,7-dimethoxycoumarin (citropten), p-coumaric acid, caffeic acid, fisetin, chlorogenic acid, 3,4-dihydroxybenzoic acid (protocatechuic acid), quercetin, quinic acid**,** and malic acid were purchased from Sigma–Aldrich (Madrid, Spain). All solvents used for the preparation of sample and mass spectrometry analysis was liquid chromatography mass spectrometry (LCMS) graded solvent purchased from Merck (Darmstadt, Germany).

The analysis was performed by using the Thermo Scientific Dionex Ultimate 3000 Series RS pump coupled with a Thermo Scientific Dionex Ultimate 3000 Series TCC-3000RS column compartments and a Thermo Fisher Scientific Ultimate 3000 Series WPS-3000RS autosampler controlled by Chromeleon 7.2 Software (Thermo Fisher Scientific, Waltham, MA and Dionex Softron GMbH Part of Thermo Fisher Scientific, Germany). Separations were performed by using a ACQUITY UPLC® CORTECS C18 analytical column (2.1 mm × 50 mm; particle size, 1.6 m) (Waters, Milford, MA, USA) equipped with a Van Guard BEH C18 pre-column (2.1 mm × 5 mm; particle size, 1.7 m) (Waters, Milford, MA, USA) maintained at 40°C. The mobile phase consisted of solutions A (0.1%v/v formic acid in water) and B (0.1% v/v formic acid in acetonitrile solution). A gradient program was used for elution including 5% solution B initially, with 5–30% solution B from 0 to 3 min, 30–90% solution B from 3 to 8 min, and 90% solution B from 8 to 11.5 min. A 4 min equilibration was adopted before the next injection. The mobile phase was delivered at a flow rate of 0.3 mL per min.

HRAMS data were acquired using a Thermo Scientific Q-Exactive Orbitrap mass spectrometer controlled by the Xcalibur 2.3 software (Thermo Fisher Scientific, Waltham, MA) and operated at 70,000 resolution in full scan and 35,000 in MS/MS scan mode. The key heat electrospray ionization (HESI) source parameters were optimized as follows: spray voltage, 3.5 kV; capillary temperature, 320 C; sheath gas flow, 30 L min1; auxiliary gas, 10 L min1 ; sweep gas, 5 L min1; heater temp, 350 C; S-lens RF level, 55%. The scope of full scan was from 50 to 500 M/Z. Data-dependent MS/MS analysis was performed with normalized collision energy of 30 V and a M/Z isolation window of 4 atomic mass units (AMU). Accurate m/z of the detected peaks were then extracted in Xcalibur (m/z window of 5 ppm) to confirm their presence. Identification of the potential detected compounds were based on their accurate m/z and observed isotopic clusters, obtained in the MS mode, as well as on their MS/MS fragmentation patterns and the accurate m/z of the resulting product ions.

### Dosing preparation

The selection of doses for this study were based on the general toxicology studies of freeze-dried CPLJ in Sprague Dawley rats, which were conducted previously by our lab [43–45]. The findings indicated that freeze-dried CPLJ was not toxic up to 2000 mg/kg as no mortality nor adverse effect was observed in rats [43–45]. The therapeutic dose was calculated based on the clinical trial study of CPLJ on dengue patients conducted previously [12]. Briefly, about 2400 mg of *C. papaya* juice powder was produced after freeze drying of 30 ml juice, which was previously consumed by dengue patients once a day for three consecutive days [12]. So, the dose consumed by a normal weight (60kg) dengue patient was calculated as 40 mg/kg BW/day. After conversion to animal equivalent dose [46], the calculated dose was 500 mg/kg BW/day. So, the dosing regimen for this study was 500 mg/kg BW once a day for 3 consecutive days. The oral treatment was done by using oral gavage on day 1 post infection to allow complete virus dissemination in the mouse circulation. The dosing volume was calculated so that it meets the 10 ml/kg bodyweight requirement. Treatment of infected AG129 mice with higher dose of freeze-dried CPLJ (1000 mg/kg BW) was also done independently using similar study design as mentioned in the previous section. In addition, the uninfected AG129 mice given 1000 mg/kg BW/day freeze-dried CPLJ alone remained healthy throughout this experiment (Fig. 4).

### Whole blood and plasma collection

The blood withdrawal was performed by submandibular vein puncture technique (facial vein), which is recommended for periodic blood collection on mice and provide a fast recovery time of the puncture wound [47]. Whole blood was collected (250 μl) in EDTA microtainer tube. The whole blood was processed for plasma collection. The blood was centrifuged for 15 minutes at 2000 x g at 4°C. The plasma was immediately transferred into a separate clean 1.5 ml microcentrifuge tube for the purpose of NS1 assay and real-time PCR virus quantitation. The plasma was stored at –40°C for future use.

### Quantitation of NS1 level in plasma

Five microliters of plasma were subjected to NS1 assay using the Platelia Dengue NS1 Ag assay kit (Bio-Rad, USA) according to the manufacturer instruction with some modifications. Briefly, 5 μl of plasma was diluted with 45 μl of phosphate buffered saline (PBS, pH 7.2). Then, 10 μl of diluted plasma was mixed with 40 μl of R7 solution and transferred into 96 well plate. Another 50 μl of R7 solution was added prior to the addition of 100 μl diluted peroxidase-conjugated NS1 monoclonal antibody solution. The mixture of sample and antibody solution were incubated for 90 minutes at 37°C. Then, the microplate was washed as described by the manufacturer. The reaction was started by the addition of chromogen solution and allowed to develop in the dark for 30 minutes at room temperature. The enzymatic reaction was stopped by the addition of stopping solution (1N sulfuric acid solution) in each well. The optical density was measured at 450 nm using a microplate reader (FLUOstar Omega, BMG Labtech, Germany).

### Detection and quantification of viral RNA

The dengue virus titer in the plasma was determined by viral RNA quantification using quantitative reverse transcription PCR (qRT-PCR), which has been shown to be more sensitive and kinetically correlated with the direct viral count by plaque assay technique [48]. The viral RNA was extracted from the plasma according to the manufacturer instruction using QIAamp Viral RNA Mini kit (Qiagen, USA). The purified viral RNA was subjected to qRT-PCR (Applied Biosystems 7500 fast, USA) as described by Chutinimitkul et. al. [49] using QuantiTect SYBR® Green RT-PCR detection kit (Qiagen, USA). The dengue viral RNA with known copy number was used as a standard for copy number determination.

### Data analysis

The ANOVA with multiple comparison test was used for comparison between groups. The differences between groups were considered significant when the *P* value was less than 0.05. The Kaplan Meier survival curves was constructed for each control and treated groups and the difference between groups was analyzed using log-rank test.

## Results

### The establishment of dengue fever model in AG129 mice

**Fig. 1:**
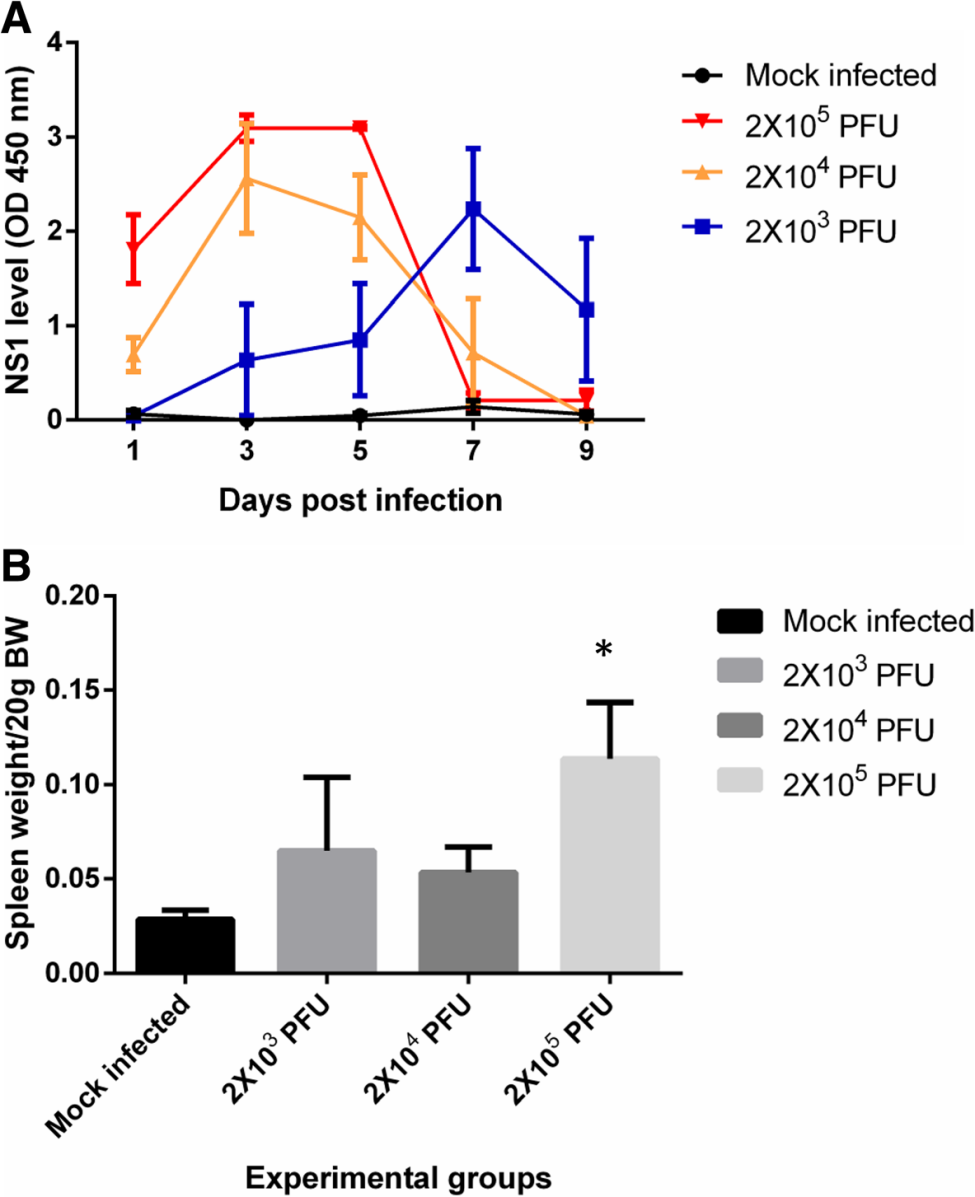
The AG129 mice infected with non-adapted serotype 2 NGC strain dengue virus developed NS1 antigenemia and splenomegaly. AG129 mice intraperitoneally infected with 2 X 10^3^ PFU or 2 X 10^4^ PFU or 2 X 10^5^ PFU of dengue virus strain NGC on Day 0 post infection. The mock infected mice group (*n*=5) were inoculated with 200 μl of media only. **a** The plasma NS1 level of mock infected (black circle) (*n*=5) and infected (*n*=5) (blue square: 2 X 10^3^ PFU; yellow triangle: 2 X 10^4^ PFU; red triangle: 2 X 10^5^ PFU) AG129 mice groups were detected on day 1, 3, 5, 7 and 9 post infection by NS1 antigen immunoassay. Data represent mean values ± standard error of the mean (SEM). **b** The spleens of mock infected (*n*=5) and infected (*n*=5) AG129 mice groups were harvested and weighed on Day 11 post infection. Data represent mean values ± standard deviations (SD). The data comparison was done by using Tukey’s multiple comparison ANOVA. Statistical significance (*p*<0.05) is depicted with asterisks above the columns

The peak of NS1 level depends on the initial viral load. The level of plasma NS1 in the AG129 mice infected with 2X10^4^ PFU and 2X10^5^ PFU peaked at day 3 post infection and start to decline from day 5 to day 7 post infection until it was not detected on day 9 post infection (Fig. 1a). In contrast, AG129 mice inoculated with lower viral load, 2X10^3^ PFU, showed a delay in NS1 production where it only peaked on day 7 post infection and declined on day 9 post infection (Fig. 1a). As expected, the NS1 level remains undetected in mock infected AG129 mice group which were only inoculated with plain media (Fig. 1a). In addition to the sign of infection, the spleen of AG129 mice infected with 2X10^5^ PFU viral load were significantly (*p*<0.05) enlarged as compared to mock infected AG129 mice group (Fig. 1b) suggesting a strong inflammatory response to dengue infection in dengue fever mouse model. No obvious spleen enlargement was observed in AG129 mice with lower viral load.

### Qualitative analysis of freeze-dried CPLJ compounds by UHPLC-HRAMS

**Fig. 2:**
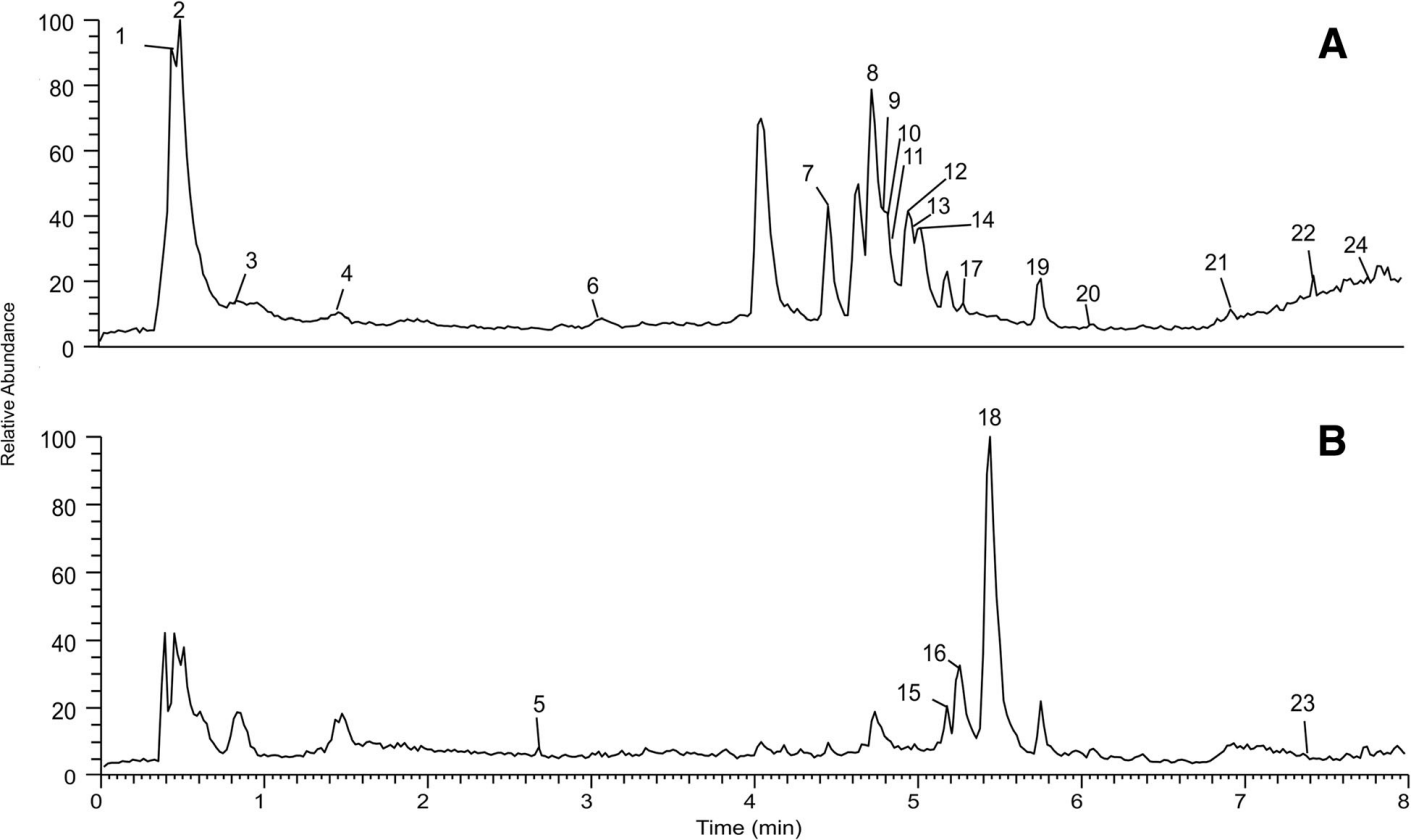
Fingerprinting of freeze-dried CPLJ by UPLC-HRAMS analysis. Chromatograms of freeze-dried CPLJ was generated by UHPLC-ESI-Orbitrap-MS/MS at (**a**) negative and (**b**) positive ionizations

Qualitative analysis was carried out on ultra-high-performance liquid chromatography coupled to ESI Q-Exactive orbitrap high-resolution accurate mass spectrometry in positive and negative modes in order to identify compounds present in the CPLJ samples. The potential calculated mass and fragmentation of the compounds associated with the measured mass of compounds were evaluated with Xcalibur software. The MS chromatograms (negative and positive ion modes) were shown in Fig. 2. Twenty-four compounds were detected in the freeze-dried CPLJ with some tentatively identified and some were identified by comparing with reference standards. Sixteen compounds such as quinic acid, malic acid, protocatechuic acid, chlorogenic acid, *p*-coumaric, caffeic acid, ferulic acid, rutin, nicotiflorin, myricetin, fisetin, morin, quercetin, kaempferol, citropten and isorhamnetin were identified based on mass spectra pattern and retention times with their reference standards. In total, 12 flavonoids, 5 hydroxycinnamic acids derivatives, 3 alkaloid compounds, 3 organic acids and 1 phenolic acid were tentatively identified and identified by reference standard in freeze-dried CPLJ as shown in Table 1.

**Table 1:** Compounds identification of freeze-dried *C. papaya* leaf juice by UPLC-HRAMS analysis

No.	Compounds	Formula	Retention time (min)	ESI Mode (Negative/Positive)	MS (m/z)/mass error (ppm)	MS/MS fragments ions (m/z)
1	*Quinic acid	C_7_H_12_O_6_	0.46	Negative	91.05545/2.28	102.94775, 127.04004 (100)
2	*Malic Acid	C_4_H_6_O_5_	0.51	Negative	133.01434/1.19	115.00343 (100), 71.01169, 89.02279, 75.00661
3	*Protocatechuic acid	C_7_H_6_O_4_	0.81	Negative	153.01880/3.69	109.02846 (100), 111.101892 (10)
4	*Chlorogenic acid	C_16_H_18_O_9_	1.41	Negative	353.08746/2.13	191.01953(100)
5	*p-Coumaric acid	C_9_H_8_O_3_	2.63	Positive	165.05493/1.87	147.04439 (100), 149.04929 (30), 119.04953 (20) 142.96738
6	*Caffeic acid	C_9_H_8_O_4_	3.08	Negative	179.03476/2.26	135.04527 (100), 136.98357 (10)
7	**Manghaslin	C_33_H_40_O_20_	4.66	Negative	755.20654/4.79	300.02884 (100), 301.03391 (40), 271.02570, 151.00323
8	**Clitorin	C_33_H_40_O_19_	4.74	Negative	739.20807/0.09	284.03326 (100), 285.03860 (50), 255.09275, 151.00337
9	**Sinapic acid	C_11_H_12_O_5_	4.77	Negative	223.06154/1.44	164.04741 (100), 149.02414 (80), 208.03912 (70), 170.07121 (20)
10	**Isoquercetin	C_21_H_20_O_12_	4.83	Negative	463.09009/2.99	300.02841 (100), 301.03516 (60), 271.02573, 402.06155
11	*Ferulic Acid	C_10_H_10_O_4_	4.89	Negative	193.05016/3.24	134.03703 (100), 178.02638 (30), 149.06056 (10)
12	*Rutin	C_27_H_30_O_16_	4.94	Negative	609.14673/2.82	300.02887 (100), 301.0583 (80) 302.03983, 151.00136
13	**Astragalin	C_21_H_20_O_11_	4.99	Negative	447.09494/2.75	284.03329 (100), 285.03998 (80), 255.03046 (20), 327.05276,
14	*Nicotiflorin	C_27_H_30_O_15_	5.04	Negative	593.14807/2.65	238.18059 (100), 256.191689 (20),137.09636
15	**Dehydrocarpaine II	C_28_H_48_N_2_O_4_	5.20	Positive	475.35425/2.34	284.03308 (50), 285.03891 (100), 273.11279, 151.00275
16	**Dehydrocarpaine I	C_28_H_48_N_2_O_4_	5.27	Positive	477.37003/2.82	240.19635 (50), 220.16989, 256.19165
17	*Myricetin	C_15_H_10_O_8_	5.31	Negative	317.03104 /4.82	178.9979 (80), 151.00313 (60)
18	**Carpaine	C_28_H_50_N_2_O_4_	5.45	Positive	479.38556/2.56	240.19623 (100), 241,19969, 222.18558
19	*Fisetin	C_15_H_10_O_6_	5.78	Negative	285.0405/3.98	163.00359 (40), 135.00841 (30) 121.0912 (20)
20	*Morin	C_15_H_10_O_7_	6.10	Negative	301.03564/4.52	151.00308 (100), 229.05057 (20)
21	*Quercetin	C_15_H_10_O_7_	6.94	Negative	301.03571/4.75	151.00320 (100), 178.99736 (50), 273.04007 (20), 257.04529 (20)
22	*Kaempferol	C_15_H_10_O_6_	7.47	Negative	285.04041/3.67	151.00298 (100), 229.9550
23	* Citropten	C_11_H_10_O_4_	7.78	Positive	207.06567/2.34	192.04166, 163.07509 (100)
24	*Isorhamnetin	C_16_H_12_O_7_	7.84	Negative	315.0517/4.34	300.02762 (100), 151,00200 (10)

*Compounds identified tentatively and confirmed with reference standards

**Compounds identified tentatively

### Effect of freeze-dried CPLJ on NS1 and viral RNA levels during dengue virus infection in AG129 mice

**Fig. 3:**
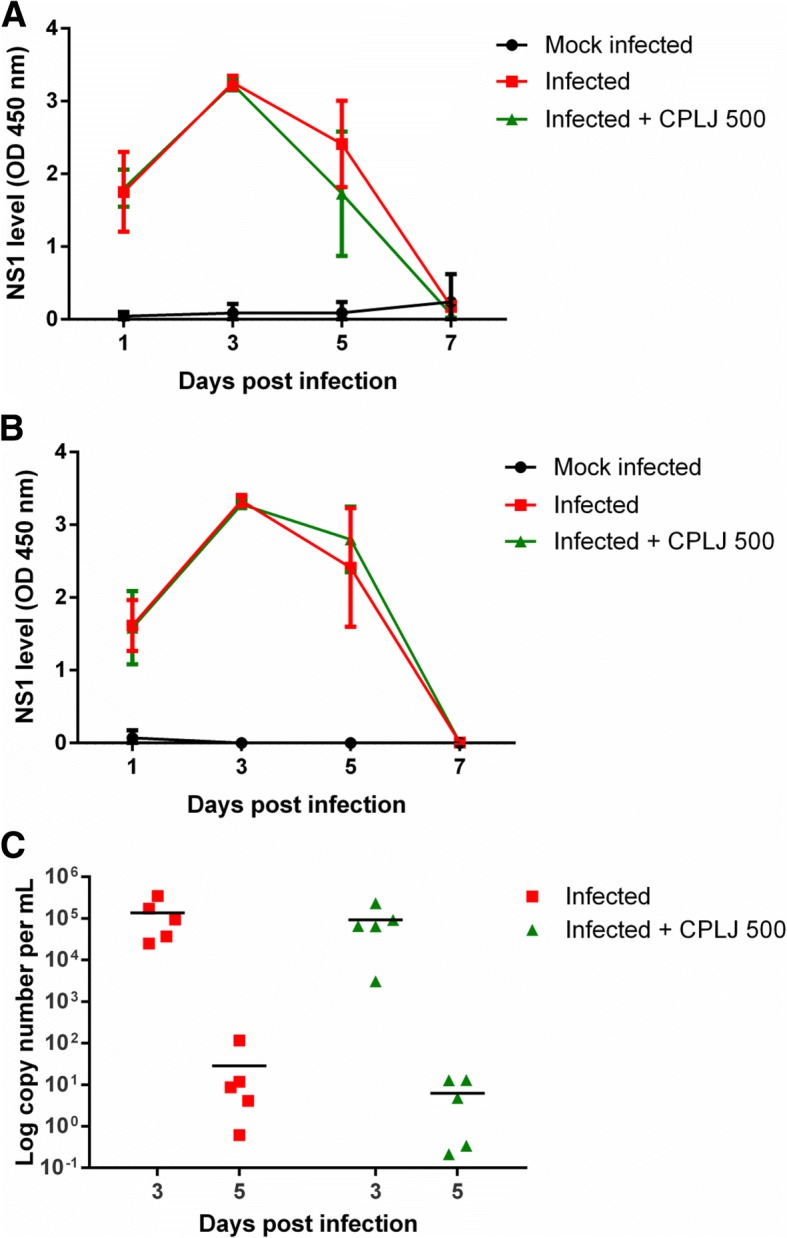
The effect of freeze-dried CPLJ on the level of NS1 and viral RNA. AG129 mice inoculated with (**a**) 2X10^5^ PFU and (**b**) 2X10^6^ PFU dengue virus strain NGC were treated with 500 mg/kg BW of freeze-dried CPLJ once a day from day 1 to day 3 post infection. The NS1 levels in plasma of mock infected (black circles) (*n*=5), infected (red squares) (*n*=5) and infected+CPLJ 500 (green triangles) (*n*=5) AG129 mice groups were detected on day 1, 3 and 5 by NS1 antigen immunoassay. (**c**) The plasma viral RNA of infected (red squares) (*n*=5) and infected+CPLJ 500 (green triangles) (*n*=5) AG129 mice groups (2X10^6^ PFU) were detected by quantitative reverse transcription PCR on day 3 and day 5 post infections. Data represent mean values ± standard deviations (SD)

In order to test the efficacy of freeze-dried CPLJ, the treatment was conducted independently on AG129 mice infected with 2 X 10^5^ PFU and 2 X 10^6^ PFU of NGC dengue virus. The AG129 mice inoculated with higher viral titer, 2 X 10^6^ PFU, was included in this study due to the higher lethality rate as compared to the mice infected with 2 X 10^5^ PFU dengue virus (Additional file 1). The kinetic pattern of NS1 production in AG129 mice infected with 2X10^5^ PFU and 2X10^6^ PFU of dengue virus was unaffected by the oral treatment of 500 mg/kg BW freeze-dried CPLJ (Fig. 3a and b). The plasma viral RNA level of AG129 mice infected with 2X10^6^ PFU was also not affected by the freeze-dried CPLJ treatment (Fig. 3c).

### Effect of freeze-dried CPLJ treatment on the morbidity level of dengue virus infected mice

**Fig. 4:**
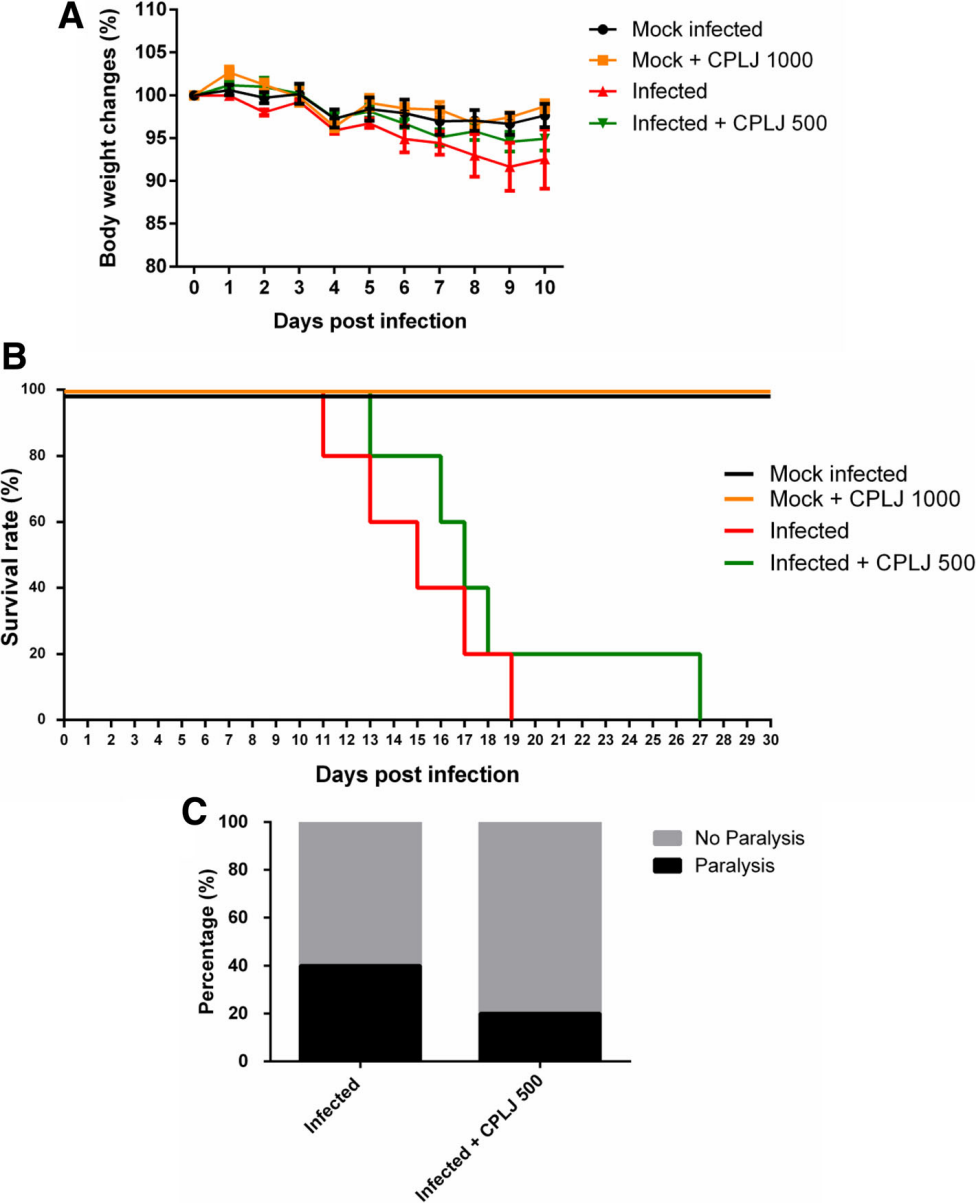
The freeze-dried *C. papaya* leaves juice slightly affecting the morbidity development of AG129 mice infected with 2 X 10^6^ PFU dengue virus. **a** Body weight changes of mock infected (black lines) (*n*=5), infected (red lines) (*n*=5) and infected+CPLJ (green lines) (*n*=5) AG129 mice groups were monitored daily. Data represent mean values ± standard error of the mean (SEM). **b** Number of moribund AG129 mice in mock infected (black lines) (*n*=5), mock+CPLJ 1000 (orange line) (*n*=5), infected (red lines) (*n*=5) and infected+CPLJ 500 (green lines) (n=5) groups were recorded daily for 30 days post infection. Kaplan-Meier curves illustrated the susceptibility of AG129 mice to dengue virus. Percentage of survival between experimental groups were compared using Log-rank (Mantel-Cox) test. However, the survival differences were not statistically significant between infected and infected+CPLJ 500 groups. **c** The number of mice developing paralysis (complete paralysis of one or both hindlimbs) were recorded for 30 days post infection. Data represent percentages of total moribund AG129 mice with and without sign of paralysis

The AG129 mice infected with 2 X 10^6^ PFU dengue virus showed the progression of the disease as the median body weight gradually decreased to more than 5% starting from day 7 to day 10 post infection (Fig. 4a). The infected AG129 mice treated with 500 mg/kg BW freeze-dried CPLJ (Infected+CPLJ 500) also showed a gradual decrease in the median bodyweight but limited to 5% on day 10 post infection (Fig. 4a). The body weight of mock infected AG129 mice group remained plateau till day 10 post infection (Fig. 4a). In addition, the mock infected AG129 mice receiving higher dose of freeze-dried CPLJ (Mock+CPLJ 1000), 1000 mg/kg BW, also showed similar body weight pattern as the mock infected AG129 mice group till day 10 post infection (Fig. 4a) suggesting that there was no sign of toxicity or illness caused by the freeze-dried CPLJ alone on the healthy AG129 mice during the experiment.

The freeze-dried CPLJ treated AG129 mice infected with dengue virus showed a slight improvement in survival rate as compared to infected AG129 mice without treatment. However, the difference was not statistically significant (p>0.05) (Fig. 4b). As neurological sign of illness is common in AG129 mice infected with dengue virus strain NGC, paralysis (complete paralysis of one or both hindlimbs) was observed in 40% of infected AG129 mice (Fig. 4c). Meanwhile, only 20% of the freeze-dried CPLJ treated AG129 mice infected with dengue virus (Infected+CPLJ 500) developed paralysis (Fig. 4c).

### The effect of 1000mg/kg BW freeze-dried CPLJ treatment on plasma NS1 and viral RNA levels of dengue virus infected AG129 mice

**Fig. 5:**
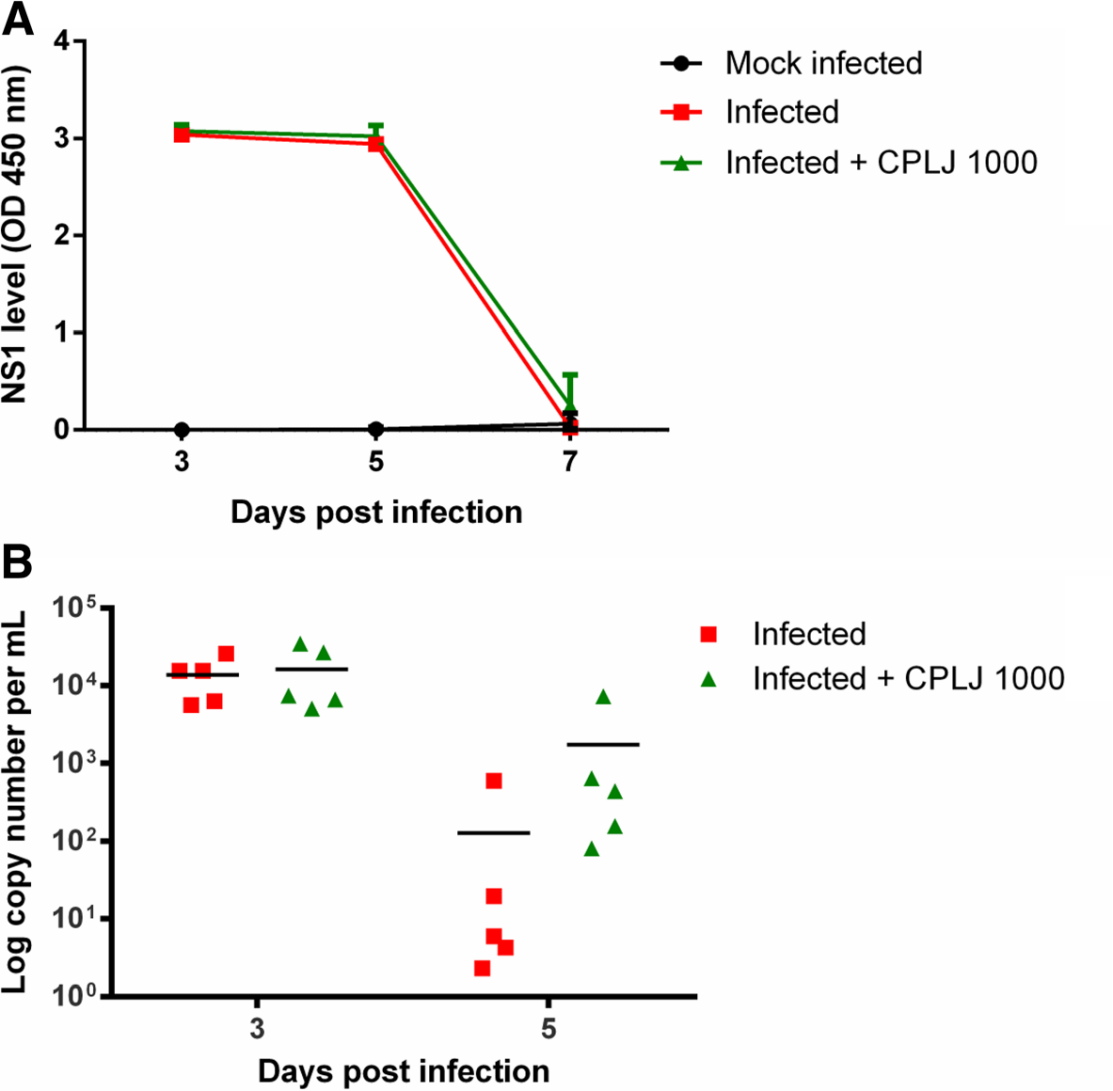
An independent experiment showing that 1000 mg/kg BW freeze-dried *C. papaya* leaf juice was not affecting plasma NS1 and viral RNA levels in AG129 mice during dengue infection. (**a**) AG129 mice infected with dengue virus strain NGC (2 X 10^6^ PFU) were treated with 1000 mg/kg BW of freeze-dried CPLJ daily from day 1 to day 3 post infection. The plasma NS1 level of mock infected (black circles) (*n*=5), infected (red squares) (*n*=5) and infected+CPLJ 1000 (green triangles) (*n*=5) AG129 mice groups were detected on day 3, 5 and 7 by NS1 antigen immunoassay. (**b**) The plasma viral RNA of infected (red squares) (*n*=5) and infected+CPLJ 1000 (green triangles) (*n*=5) AG129 mice groups were detected by quantitative reverse transcription PCR on day 3 and day 5 post infections. Data represent mean values ± SD

In order to see whether there is a dose related effect of freeze-dried CPLJ on the dengue virus development in AG129 mice, a higher dose of freeze-dried CPLJ, 1000 mg/kg BW, was used to treat the infected AG129 mice. It was clearly shown that after treatment with 1000 mg/kg BW of freeze-dried CPLJ, there was no significant difference in the NS1 level on day 3, 5 and 7 post infection as compared to mock infected AG129 mice (Fig. 5a). The plasma dengue viral RNA level was also not significantly affected by the treatment with higher dose of freeze-dried CPLJ (Infected+CPLJ 1000) (Fig. 5b).

## Discussion

The test system for this study, AG129 mice infected with non-mouse adapted DEN-2 dengue virus strain, NGC, produced antigenemia and viremia as confirmed by the presence of NS1 and viral RNA in the plasma, respectively. The test item, freeze-dried CPLJ, mainly consists of flavonoid derivatives followed by hydroxycinnamic acids derivatives, alkaloid compounds, organic and phenolic acids. However, the oral administration of freeze-dried CPLJ (500 mg/kg/day and 1000 mg/kg/day) for 3 consecutive days did not affect the level of NS1 and viral RNA in the plasma of dengue virus-infected AG129 mice.

The development of NS1 antigenemia in infected AG129 mice showed the ability of non-mouse adapted NGC strain DEN-2 dengue virus to replicates in AG129 mice, which lack of interferon α/β and interferon γ receptor genes. A similar kinetic pattern of NS1 has also been demonstrated in AG129 mice infected with other dengue virus strains, which peaked on day 3 to day 4 post infection and the concentration of NS1 in the plasma was positively dependent on the viral inoculation dose [50]. This study could not mimic the severe dengue condition because the clinical symptoms developed by AG129 mice infected with NGC strain DEN-2 dengue virus was limited to viremia and paralysis. The NGC strain DEN-2 dengue virus was commonly use in viremia development or anti-viral studies [31, 51]. There were several non-mouse adapted dengue virus strains, which represent dengue hemorrhagic fever [36, 37, 52, 53]. For example, the D2Y98P strain DEN-2 dengue virus has been reported previously to be lethal and could cause plasma leakage in infected AG129 mice in less than 10 days post infection [37, 52]. As compared to this study, the infection with non-mouse adapted NGC strain DEN-2 dengue virus was lethal at later time point (day 11 post infection onwards). As the NGC strain DEN-2 dengue virus is neurovirulent, most of the infected AG129 mice were found paralyzed at later day of post infection, which could be due to the systemic infection in the brain [27]. However, not all dengue virus strains produced thrombocytopenia in AG129 mice. This includes the NGC strain DEN-2 dengue virus used in this study and D2Y98P strain DEN-2 dengue virus [37, 52]. Thereby, this study was unable to observe any reduction of platelet level in the infected AG129 mice.

Out of 24 identified compounds, flavonoids derivatives followed by hydroxycinammic acids derivatives was majorly identified in the freeze-dried CPLJ. Similar findings were also reported by Anjum et al. [13] and Afzan et al. [44] studies, which have highlighted the abundance of flavonoids and hydroxycinammic acids derivatives in the freeze-dried CPLJ. As based on the peak area, the dominant compounds were quinic acid, malic acid, manghaslin, clitorin, sinapsic acid, isoquercetin, ferulic acid, rutin, astragalin and nicotiflorin and carpaine. The clitorin, manghaslin and carpaine were also reported as dominant compounds in the freeze-dried CPLJ of ‘Sekaki’ cultivar by Afzan et al. [44].

The potential dengue anti-viral compounds such as quercetin and fisetin were among the identified compounds in the freeze-dried CPLJ. The dengue anti-viral activities of quercetin and fisetin were detected by *in vitro* studies with 50% inhibitory concentrations (IC_50_) of 35.7 μg/ml and 55 μg/ml, respectively [26, 54]. However, as the amount of quercetin and fisetin in the freeze-dried CPLJ could be low, the antiviral activities of these compounds may be hindered or not biologically effective *in vivo*. In addition, the doses and treatment duration used in this study might not be sufficient to inhibit dengue virus replication *in vivo*. This is because the doses and treatment duration as implemented in this study (therapeutic dose: 500mg/kg/day and high dose:1000 mg/kg/day; 3 consecutive days) were based on the clinical trial conducted for the assessment of thrombocytosis activity [12] but not for its antiviral activity. Therefore, in order to better understand the effective anti-viral doses and treatment duration of freeze-dried CPLJ, a pharmacokinetic study for at least 3 different doses (low, medium and high doses) need to be conducted in the future.

Although freeze-dried CPLJ treatment was not affecting the plasma viral RNA level, the potential effect of freeze-dried CPLJ on the organ’s viremia could not be excluded. However, due to the nature of this study (observational study), the possible effect of freeze-dried CPLJ treatment on dengue virus viability in the organs such as liver, spleen, kidney and brain could not be highlighted. While dengue patients are commonly viremic at the time of treatment, studying the effect of *C. papaya* juice at different phases of dengue clinical development, febrile phase (early infection, peak viremia) and critical phase is important to discover the optimal administration of the juice in clinical settings.

The dengue fever mouse model in this study was not exactly representing the patient’s condition in the clinical setting as reported in previous studies [10–12]. Other than the viremic condition of dengue patients, the enrolled dengue patients in *C. papaya* juice clinical trial were treated with *C. papaya* leaf juice together with the implementation of standard dengue management such as hydration and paracetamol treatments. Hence, whether or not, the *C. papaya* leaf juice exhibit synergistic effect when used in combination with the standard dengue management (at least with paracetamol combination) in dengue mouse model and how this combination therapy affects the dengue virus development remains to be elucidated.

## Conclusion

The findings indicated that 3 days treatment of 500 mg/kg/day and 1000 mg/kg/day of freeze-dried CPLJ at early point of infection or at the onset of viremia (Day 1 to Day 3 post infection) did not affect the plasma NS1 and viral RNA levels in dengue fever AG129 mice model. More studies need to be conducted in order to validate the findings, with special attention to the dose, the duration of treatment, and potential use of CPLJ in adjunctive therapy.

## Additional file

Additional file 1: The survival rate of AG129 mice infected with 2 X 10^5^ PFU and 2 X 10^6^ PFU NGC strain dengue virus. (PDF 29 kb)
