# Supplementary material for: The effect of freeze-dried Carica papaya leaf juice treatment on NS1 and viremia levels in dengue fever mice model
Source: BMC Complement Altern Med. 2018 Dec 5;18:320. doi: 10.1186/s12906-018-2390-7 (PMC6282281; doi:10.1186/s12906-018-2390-7)
Supplement: Supplementary file 1 — The survival rate of AG129 mice infected with 2 X 105 PFU and 2 X 106 PFU NGC strain dengue virus. (PDF 29 kb) [file 12906_2018_2390_MOESM1_ESM.pdf]

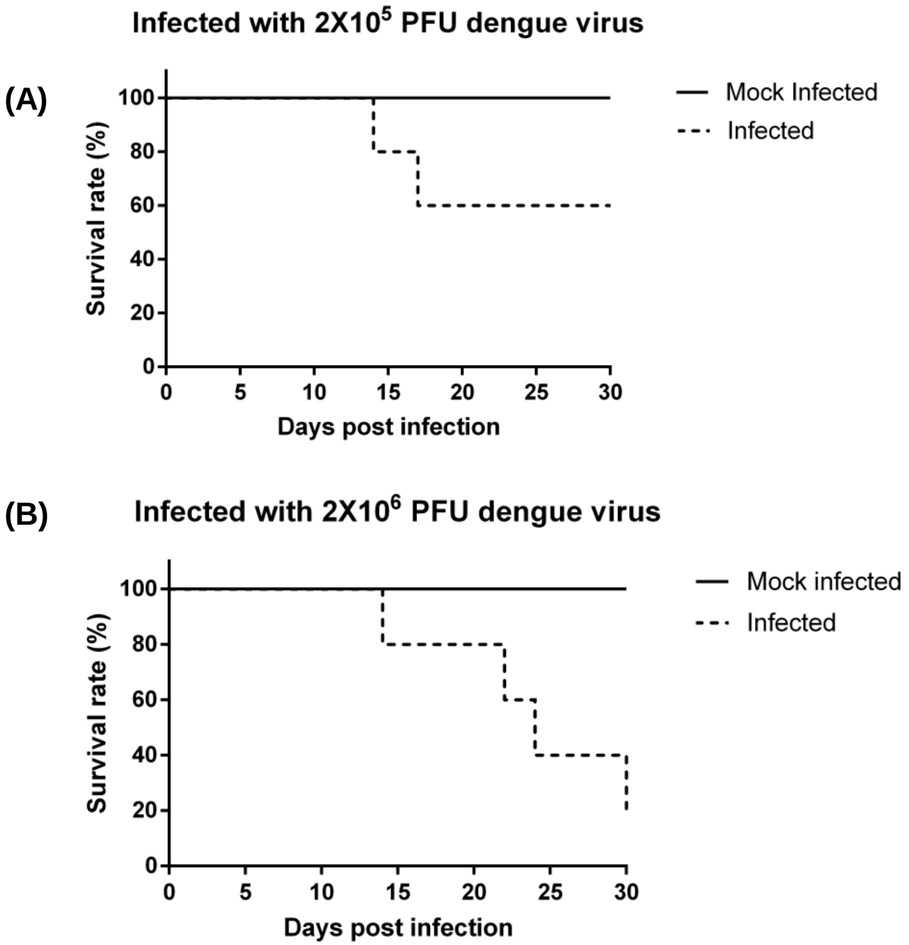

Additional file 1: The survival rate of AG129 mice infected with 2 X 10^5 PFU (A) and 2 X 10^6 PFU NGC (B) dengue virus.
